# Returning to care after incarceration with HIV: the French Guianese experience

**DOI:** 10.1186/s12889-020-08772-9

**Published:** 2020-05-24

**Authors:** F. Huber, S. Vandentorren, A. Merceron, T. Bonifay, A. Pastre, A. Lucarelli, M. Nacher

**Affiliations:** 1 COREVIH Guyane, Centre Hospitalier Andree Rosemon, Cayenne, Guyane Française France; 2 Réseau Kikiwi, Cayenne, Guyane Française France; 3 Département d’épidemiologie sociale, INSERM, Sorbonne université, Institut Pierre Louis d’Epidémiologie et de Santé Publique (IPLESP), Paris, France; 4 Université des Antilles et de la Guyane, Faculté de Médecine Hyacinthe Basturaud, Pointe-à-Pitre, France; 5 UCSA, Centre Hospitalier Andree Rosemon, Cayenne, Guyane Française France; 6 Hôpital de Jour Adulte, Centre Hospitalier Andree Rosemon, Cayenne, Guyane Française France; 7 Inserm CIC Antilles-Guyane INSERM 1424 (Pole Guyane), Universite de Guyane, Cayenne, France

**Keywords:** HIV, Prison, Antiretroviral therapy, Ambulatory care

## Abstract

**Background:**

HIV prevalence in correctional facilities may be 2 to 10 times higher than in the general adult population. Antiretroviral therapy (ART) interruption is frequent after an incarceration. This, in combination with post-release high-risk behaviors, may have detrimental consequences on the epidemic. Although return to care after release from correctional facilities has been described in many North American settings, data from South America seemed scarce.

French Guiana is the only French territory located in South America. In 2014, HIV prevalence was estimated at 1.2% among pregnant women and oscillated around 4% in the only correctional facility.

**Method:**

HIV-infected adults released from the French Guiana correctional facility between 2007 and 2013 were included in a retrospective cohort survey. The first objective was to describe the cascade of care in the 4 years following release. The secondary objectives were to describe contacts with care and to identify factors associated with return to HIV care, 1 year after release.

**Results:**

We included 147 people, mostly males (81.6%). The median time before the first ambulatory consultation was 1.8 months. Within 1 year after release, 27.9% came for unscheduled emergency consultations, 22.4% were hospitalized. Within 4 years after release, 40.0–46.5% were in care, 22.4% archieved virological success.

Being on ART when incarcerated was associated with HIV care (aIRR: 2.0, CI: 1.2–3.0), whereas being HIV-diagnosed during the last incarceration was associated with poor follow-up (aIRR: 0.3, CI: 0.1–0.9).

**Conclusion:**

The risk of HIV-follow-up interruption is high, after an incarceration with HIV. ART supply should be sufficient to cover the timespan following release, several months if possible. Those not on ART at the time of incarceration may require special attention, especially those newly HIV-diagnosed while in custody. Comprehensive programs are necessary to support ex-offenders to stay on ART after incarceration.

## Background

People in prisons and other closed settings are identified as key populations for HIV risk acquisition by WHO. Indeed, HIV prevalence in correctional facilities may be 2 to 10 times higher than in the general adult population [1]. With more than 11 million people incarcerated in 2016, the most vulnerable to HIV acquisition are over-represented in correctional facilities [2, 3]. Post-release high-risk behaviors, combined with antiretroviral therapy (ART) interruption, may have a strong impact on the general population, thus, keeping HIV under control after incarceration is strategic from a public health perspective [4, 5].

French Guiana is the only French territory located in South America, between Surinam and Brazil. In 2014, HIV prevalence was estimated at 1.2% among pregnant women and oscillated around 4% in the only correctional facility. In French Guiana’s prison, HIV prevalence is around twice the prevalence of the French mainland prisons, and around four times the prevalence in the region. We estimated that 4.5 to 5.0% of people living with HIV (PLWHIV) experienced one incarceration or more between 2007 and 2013 [3].

Recently, we pointed that the socioeconomic situation of HIV-infected inmates was particularly precarious, relative to other detainees [3], and that the male standardized mortality ratio after release was very high, reaching 14.8 times the age-specific mortality rates for males in French Guiana [6].

Although retention in HIV care after release from correctional facilities has been described in many North American settings, data from South America seems scarce [7]. The situation in French Guiana was hypothesized to be singular and warrant investigation because of the low density of health professionals, the poverty of a large segment of the population, and the intense migratory movements between low and middle income HIV-endemic countries and this French territory with half of the adult population being foreigners. Moreover, although some studies in the USA have suggested benefits for some interventions to improve return to care, a large randomized control trial failed to show any difference between traditional case management and transitional case management interventions [8, 9]. This suggest that there is a need to identify people with the highest risk of falling out of care in order to target them for comprehensive interventions. .

To better understand the prognosis and outcomes of people living with HIV released from French Guiana’s correctional facility, we conducted a retrospective cohort survey.

The first objective of this study was to describe the linkage to HIV care and the cascade of care in the 4 years following release.

Secondary objectives were to describe the contact with health care and to study the factors associated with return to HIV care, 1 year after release.

## Methods

### Study setting

French Guiana’s sole correctional facility includes a prison for males (« centre de détention », for persons sentenced for more than 2 years), a jail for males and a jail for women (« maison d’arrêt », for persons waiting for trial or sentenced for a duration shorter than 2 years) [6].

HIV testing and ARV treatments are proposed to any incarcerated patients, according to the French National recommendations [10]. Before the last recommendation issued at the end of 2013, the threshold to start ART was 500 CD4 cells/mm3 [11]. After HIV positive confirmation, all HIV-positive patients were registered in the electronic medical record NADIS, used at the regional level for HIV-follow-up, and gave written consent for electronic data entry, data analysis, and research publication. NADIS on-line software was shared and available for any HIV specialist working in French Guiana.

After release, the PLWHIV were given a follow-up appointment in a specialized care unit, usually in one of the 3 main hospitals of the territory, within 7 days following release. They received a one-week provision of ART.

### The DAI-VIH retrospective cohort

The DAI-VIH retrospective cohort was conducted among all the HIV-infected adults (> 18 years old) released between 1/1/2007 and 12/31/2013, after an incarceration of 1 month or more. The last incarceration of the time period was defined as incarceration of reference [6].

The main source of data was the electronic medical records NADIS. Additional data were collected from the correctional facility paper files, the registries of the three regional hospitals, and the municipal death registries of the main towns of French Guiana (covering 80% of the French Guianese population). Identification relied on name and date of birth and was performed by a medical doctor. Data were recorded manually and anonymized, by the same person.

The data collected were socio-demographic characteristics, number of past incarcerations, date of entry and release of the index incarceration, medical and psychiatric background, substance abuse, HIV history, comorbidity, HIV-clinical stage, opportunistic infections, immunological and virological results during the index incarceration, post-release events: consultation in HIV care units, unscheduled emergency consultations, hospitalizations, occurrences of opportunistic infections or any CDC-stage C events, further incarcerations, date and cause of death.

“Returned to HIV care” was defined as being in care for HIV after release, whether it was in an ambulatory unit, or following a hospitalization or a new incarceration. “Undetectable viral load” was defined as an HIV viral load (VL) < 50 copies/ml. Patients were considered as “in HIV care” within a period of time, if an undectectable VL was documented, whether a HIV follow-up consultation was registered or not during the same interval. More detailed information (methodology of the DAI-VIH cohort, duration of the index incarceration, characteristic of the study population …) have already been published [6].

We used Poisson regression with robust variance, as this method is a good alternative for the analysis of cross-sectional studies with binary outcomes, with resulting estimates close to those obtained from the Mantel Haenzel procedure [12]. Covariable were included in the final model after bivariate analyses, if *p* < 0.2 Statistical analyses were performed with Stata 11 (Stata Corporation, college station, Texas, USA).

The protocol was approved by the Ethics Committee of Cayenne Hospital, and the data base was declared to the Commission Nationale de l’Informatique et des Libertés (CNIL), under the number 1975083v0.

## Results

All the PLWHIV with the inclusion criteria were included in the DAI-VIH cohort (*n* = 147). They had an observational period of one to 7 years after release. The median time since the index incarceration was 9 months.

The majority were males (81.6%), migrants (68.7%), with a median age at 37.3 years (mean: 37.8, CI: 36.3–39.2). The socio-economic status was low, 25.8% were homeless and 34% were crack-cocaine users. Most patients had an early stage HIV infection (78.1% CDC- stage A). On release, 50.3% were on ART. Reasons for not being treated were not fulfilling the criteria for 74.6% and refusing for 15.1%. Among those treated, 81.3% were virologically suppressed (viral load< 50 copies/mL) [6].

### Return to HIV care after release

As seen Tables 1, 70.1% of the included patients returned to HIV care after release. It was in an ambulatory department for 51.0% (*n* = 75), or after being hospitalized (12.2%, *n* = 18), or re-incarcerated (6.1%, *n* = 9). No consultation with an HIV physician had been documented for 29.9% of the formerly incarcerated persons.

**Table 1:** Factors associated with being in HIV care at 12+/− 3 months, after release from French Guiana correctional facility (*n* = 132)

	HIV care (%)		Crude IRR (95%CI)	p	Adjusted IRR (95%CI)	p
	Yes (*n* = 59)	No (*n* = 73)				
**Chronology between ART and incarceration**						
- No ART on release	25.7	74.3	Reference		Reference	
- ART started/restarted in prison	50.0	50.0	1.9 (1.1–3.4)	0.02	1.4 (0.8–2.4)	0.20
- On ART while incarcerated	79.4	20.6	3.1 (2.0–4.8)	< 0.01	2.0 (1.2–3.0)	< 0.01
Advanced HIV disease*	67.3	32.7	2.1 (1.5–3.1)	< 0.01	1.5 (1.0–2.1)	0.04
**Chronology between 1st positive HIV-test and incarceration**						
- Diagnosed outside custody	63.6	36.4	Reference		Reference	
- Diagnosed in custody, before the last incarceration	42.0	58.0	0.7 (0.4–1.0)	0.03	0.8 (0.6–1.2)	0.35
-Diagnosed during the last incarceration	11.1	88.9	0.2 (0.5–0.8)	< 0.01	0.3 (0.1–0.9)	0.03

*Defined by: previous AIDS illness (CDC stage C), or CD4 nadir < 200/mm3

Cofactors tested but not retained after final analysis (*p* > 0·20) were: addiction, duration of the index incarceration, homelessness. Cofactors retained in the final analysis (*p* < 0.20) but not in the final model were: sex, re-incarceration, co-morbidity, psychiatric background, native from Surinam

For those who linked to the ambulatory HIV department at any time, (n = 75), the median time between release and first consultation was at 1.8 months (mean: 6.7, inter quantile range (IQR): 0.5–8.2). It was shorter for patients on ART at release: median 1.5 months (mean: 3.5, IQR: 0.4–2.6), compared to PLWHIV released without ART: median 9.1 month (mean 13.8, IQR: 2.0–16.9).

Among the 147 included persons, in the 90 days following release, 36.1% (53/147) returned to HIV care, 31.3% declared to be on ART (46/147), 12.9% had a documented undetectable viral load (19/147).

As seen in Fig. 2, over a period of 4 years (+/− 3 months), the proportion of patients in HIV care fluctuated between 40.0 and 46.5% after M6 (+/− 3). After the first 6 months, this was relatively stable over time, as for those who declared to be on ART, which fluctuated between 35.0 and 36.8%.

**Fig. 1:**
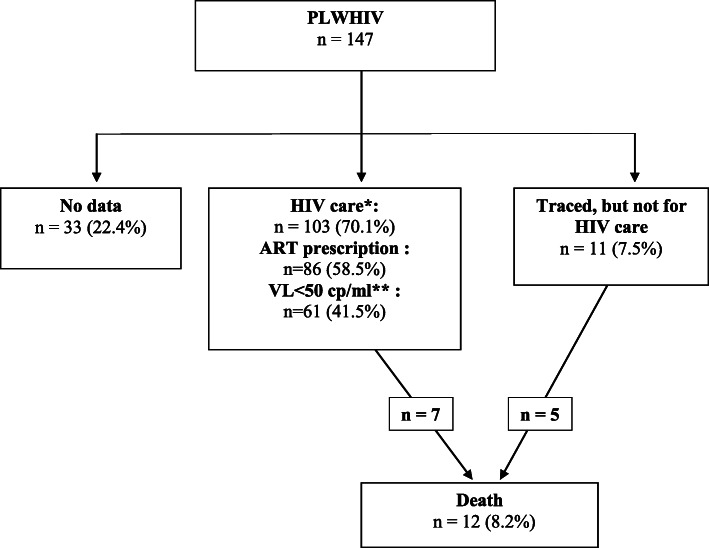
Flow of patients, following release from the index incarceration. *Whether it was in an ambulatory HIV unit (*n* = 75), after been hospitalized (*n* = 18), or during a new incarceration (*n* = 9). **At least once within the post-release follow-up period

**Fig. 2:**
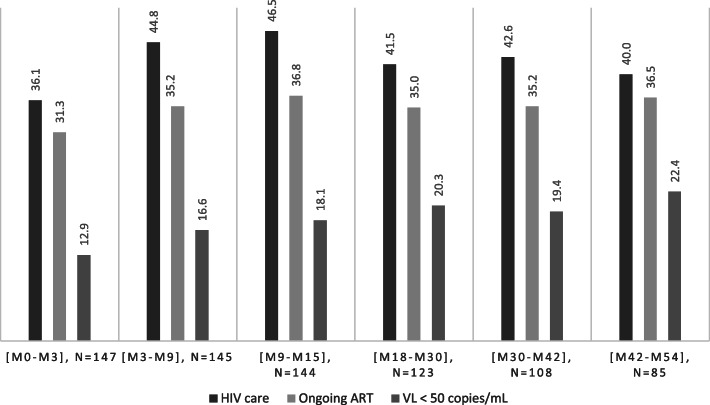
Cascade of care in the 4 years following the release from the index incarceration

Overall, 41.5% (*n* = 61) of patients reached an undetectable viral load (Fig. 1) at any time during the post-release period, but the viralogical control was not stable over time for many of them, explaining why the proportion in success for each interval was far below this number. Nevertheless, virological control seemed to be slowly improving, reaching 22.4% of the cohort, 4 years after release (Fig. 2).

Restricting the analysis to those who declared to be on ART in the interval, the proportion virologically suppressed was at 49.1% at 1 year, 58.1% at 2 years, 55.3% at 3 years and 61.3% at 4 years.

### Contact with the health care system, excluding HIV care

Whether followed in specialized care or not, a high proportion of patients had contacts with hospital care. Thus, at 12 +/− 3 months following release, 27.9% (*n* = 41) had an unscheduled emergency consultation, 22.4% (*n* = 33) had been hospitalized, and 7.5% (*n* = 11) were diagnosed with an opportunistic infection. Besides that, 10.9% (*n* = 16) were reincarcerated and 2.7% (*n* = 4) died. Within the 2 years following release, 34.1% (42/123) had an unscheduled emergency consultation.

### Return to care 1 year (12 +/− 3 months) after release

**Fig. 3:**
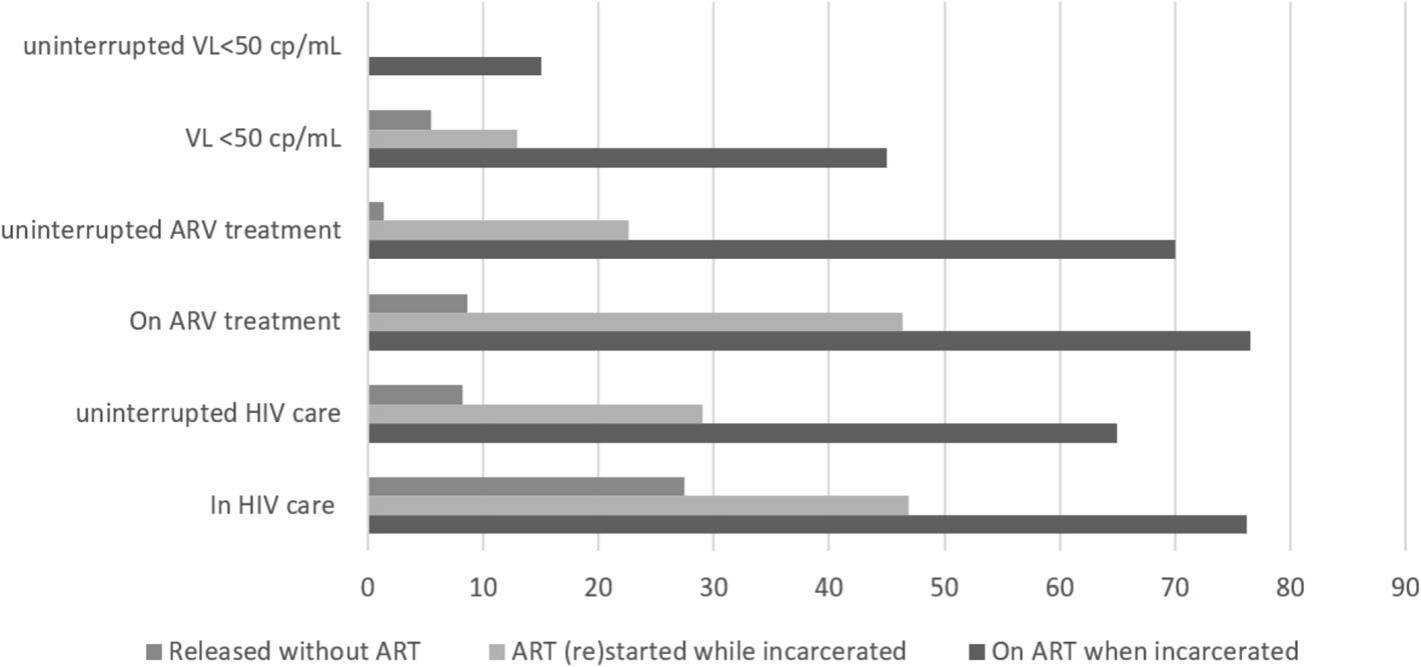
Care events, 1 year (+/− 3 months) after release from the French Guiana correctional facility (%, *N* = 144). « Uninterrupted »: at least one event in the 3 months following release, plus one event at M6+/− 3, plus one event at M12+/− 3 Re-incarcerated and loss to follow-up people were included, but not deaths.

As seen in Fig. 3, between 70 to 80% of people already on ART when incarcerated were in care at 1 year +/− 3 months and were on ART without interruption. A few patients among the latter came to renew their prescription without any medical consultation or lab test. Indeed, only 15% had a documented uninterrupted viral load < 50 copies/mL, the majority of those not on ART when incarcerated had no medical follow-up and were not on ART at 1 year +/− 3 months after release, even if taking ART in custody. The latter had frequent care or treatment interruptions.

After excluding re-incarcerated patients, and those who died, 44.7% (59/132) returned to HIV care at 1 year, 25.7% (18/70) among those released without ART, 50.0% (14/28) among those who start or restarted ART while incarcerated, and 79.4% (27/34) among those already on ART when incarcerated (*n* = 132). Only a minority of patients released without ART declared to be on ART (*n* = 6) or were virologically suppressed (*n* = 3) after release.

We explored the factors associated with being in care 1 year after release, excluding those who died and those re-incarcerated within this time-period (n = 132).

As seen in Table 1, ART was the strongest factor associated with linkage to care: patients already on treatment while incarcerated had the best outcomes (adjusted IRR: 2.0, CI:1.2–3.0), followed by those for whom ART was introduced or re-introduced while incarcerated, although the latter group variable was no longer significant in the multivariate analysis (aIRR: 1.4, CI: 0.8–2.4).

Being diagnosed with an advanced HIV-infection was also associated with return to care (aIRR: 1.5, CI: 1.0–2.1), whereas being HIV-diagnosed during the last incarceration seemed associated with care discontinuation (aIRR: 0.3, CI: 0.1–0.9). Factors tested but not retained in the final model were: age, sex, country of birth, drug or alcohol consumption, psychiatric disorder, co-morbidity, homelessness, previous incarceration, and duration of the index incarceration.

## Discussion

### Long delay to return to HIV-care, despite frequent unscheduled emergency consultations

Patients on ART left prison with a 1-week supply and returned to care after a median 45 days, we thus assume that a vast majority had interrupted treatment.

Significant delays until the first HIV-ambulatory care visit has also been reported in North American studies [13, 14]. Seeking for ambulatory care may not be the priority for formerly incarcerated persons. In addition to significant psychosocial barriers to care [3, 6, 13, 15, 16], they may also have different representations and attitudes towards health, time and forward planning for consultations. Thus, drug shortages are frequent [14, 17, 18].

Longer ART provision has been delivered in French Guiana, in the light of our data. Ideally several months of supply should be delivered, using single-tablet regimens with a high genetic barrier therapy [19].

In our survey, around one third came for an unscheduled emergency consultation within 2 years after release. Despite the poor retention in HIV-care, formerly incarcerated persons have frequent contacts with hospital care for emergency purposes, often in relation to homelessness and recent drug use [13, 20]. Ideally, emergency departments should be strategic places to identify and refer lost-to-follow-up PLWHIV.

### Poor retention in care and lost benefits after release, an old issue

For those treated in correctional facilities, viral suppression on ART is generally observed, sometimes surpassing those in the non-incarcerated population. Nevertheless, virological suppression is often not sustained after release [3, 7, 18, 19, 21], mortality is high [6, 22] and retention in care is poor [17, 23–25].

The proportion of patients who returned to care at 90 days post-release was of similar magnitude (36.1%) than in the Texas department of criminal justice (28%) [24], in Dallas country jail (34%) [26], in the Philadelphia prison system (29.4%) [27]. In contrast, in Connecticut (60.7%) [25], Rhode Island (43%), and North Carolina (49%) [14], the proportion of persons returning to care was higher than in French Guiana.

Six months after release, the USA 10-multisite EnhanceLink Initiative reported a sustained retention in care for 38%, *sustained retention* being defined as having a clinic visit during each quarter in the 6 month post-release period [23]. In Wisconsin, 67% of PLWHIV were linked to HIV care within 180 days in an recent observational survey [28].

One year after release, 24.7% of the formerly incarcerated HIV-positive persons were in care in the Philadelphia prison system [27], 67% in Connecticut [29]. Our result was in between, at 45.8%.

Although most surveys reported early return to care indicators, at 90 days or less, we assume that data at 6 months or more may be more relevant, given delays to return: in French Guiana, the proportion in HIV care was 36.1% at 3 month, and 44.8% at 6 months.

### Factors associated with return to HIV care

It has been shown that poor retention in HIV care predicts poor survival [30, 31], thus, it is unsurprising to find a poor retention, and a high rate of mortality among former HIV-infected [6, 22].

Some risk factors identified in our study were consistent with previous surveys: being diagnosed with an advanced HIV-infection was correlated with returning to care at 1 year, in our survey (aIRR: 1.5, CI: 1.0–2.1) whereas HIV-only status was a predictor of low retention in a North Carolina statewide study [31].

Like in French Guiana, receiving ART while incarcerated was associated with return to care in Texas [24] and Connecticut [29]. Other surveys showed that receiving ART in custody was associated with early linkage and retention in care [24, 25, 32]. However, the impact may differ, between patients on ART when incarcerated, and those starting or restarting ART in prison. In our experience, and perhaps other’s, most of the latter were not in HIV care 1 year after release [26].

Hence patients *not on ART* when incarcerated may require greater attention and support for release preparedness and linkage to care.

In addition, receiving the HIV-diagnosis in custody negatively impacted the linkage to care 1 year after release in our setting. Although this has not been explicitly described elsewhere, it is in line with previous surveys [33]. In contrast, prior HIV clinic follow-up before incarceration was a strong predictor of linkage to care within 90 days of release from Dallas country jail [26], and those not previously in care had longer linkage times to care in Rhode Island and North Carolina [14].

Being HIV-diagnosed while incarcerated may be a proxy for a behaviorally distinct group of patients, notably less health-conscious, or less likely to adhere to the constraints of scheduled care. People HIV-diagnosed in custody may also be more marginalized and socially vulnerable than others (in our experience, they are more often migrants [3]).

Besides that, diagnosis in Prison may impact the post-release relation to care. In French Guiana, HIV ambulatory units offer a comprehensive package of care, including counselling, health education, psychological support, by experienced multidisciplinary team (nurses, social workers, psychologist) where newly diagnosed people are repeatedly seen. The situation is different for those diagnosed in correctional facility, often left alone to cope with the shock and distress after receiving the HIV diagnosis. Indeed, patients frequently describe custody as a hostile environment, with concerns about confidentiality and lack of friendly support. As elsewhere, HIV-related stigma segregation are common [34]. Furthermore, newly diagnosed persons may be reluctant to consult an unknown ambulatory unit after release.

Unlike other studies [26], psycho-social vulnerability factors like drug use and homelessness, were not associated with poor return to care in our survey. This may be due to our methodology, as we decided to conduct multivariate analysis after excluding people reincarcerated within the year following release.

Indeed, multiple incarcerations are often associated with drug-use and homelessness [6, 35]. Re-incarcerations may have a protective effect for the triple burden subpopulation “homeless-addicted-multi-incarcerated”, through access to care and re-initiation of ART while incarcerated [25]. With our methodology, we probably underestimate the effect of the “homeless-addicted-multi-incarcerated” subpopulation.

Minorities, like black and latinos had sometimes poorer outcomes in terms of return to care [14, 36], although this is controversial [26]. We were not able to investigate these factors as racial statistics are not allowed in the French Law. Nevertheless, we found no impact of the country of birth after multivariate analysis.

### Improving the linkage to care of formerly incarcerated HIV-positive patients

In 2001, through the experience of the Project Bridge Program held in Rhode Island (USA), Rich stated that formerly incarcerated HIV-positive persons could stay in care when given adequate support [37]. Since then, observational data showed that many interventions were efficient: transitional case management [25, 38, 39], discharge planning [23, 24, 40], provision of post-release housing [40], HIV education [23], transportation assistance [23], navigation program [28].

Some recent interventions seemed to have favorable impacts: patient navigation program combined with enhanced case management [36], peer navigation intervention [41], telephone contact by a continuity clinic coordinator [42]. However, a recent large study did not show any difference between routine discharge planning and the ImPACT intervention [8, 9]. Thus there is a need to identify people at highest risk of interrupting care after release to target them for comprehensive interventions [8].

Given the similarity of many of our results to those described in the USA, we could implement these US experiences, and advocate to reinforce the five necessary components described by Springer and al to improve the transition to community care: case management services, continuity of ART, treatment of the substance use disorder, continuity of treatment for mental illness, and reducing HIV-associated risk-taking behavior [16]. Furthermore, maintaining contact with care givers through cellphone provision, may be an affordable pragmatic intervention to test in French Guiana.

### Limitations and strengths of our study

The small sample size, and the retrospective nature of the study are limitations. Information recorded from the medical files may have been interpreted inaccurately, as it was not standardized. Besides this, non-medical confounding factors (like health coverage, educational status …) were not always available. We cannot exclude a bias due to loss of follow-up as well, as we found no information for 22.4% of the patients after their release. As they were mostly migrants, some may have returned to care in a neighboring country.

Furthermore, our study cannot pretend to explain the overall dynamics of return to care at different time points. Indeed, some factors associated with HIV care at 12 months differed when the outcome was uninterrupted care (Supplementary table).

The strength of our cohort is that with 56.5% of persons initiating ART in prison there was a balance between pretreated and non-pretreated persons, which seemed to be an interesting segmentation in terms of outcome, a distinction that is usually absent from other studies [6].

## Conclusions

Despite the virological benefits while incarcerated for those on ART, a high rate of formerly incarcerated HIV-positive persons released from the French Guiana Correctional facility died soon after release [6] and return to care was poor.

Our study highlights some key facts already described the North American surveys, which may lead to pragmatic recommendations.

Indeed, test and treat strategy may have a strong effect for the retention in care for formerly incarcerated HIV-positive persons and may be advice whenever feasible.

Formerly incarcerated persons take time to return to ambulatory HIV care, meaning a high risk of drug shortage.

Formerly incarcerated persons may come frequently to the hospital for unscheduled emergency consultations. Identifying and referring these patients back to ambulatory HIV care may improve return to care after release.

Being already on ART at the time of incarceration seems strongly associated with returning to care after release; those *not on ART* when incarcerated may require more support than others, as well as those newly HIV-diagnosed in custody.

## Supplementary information

**Additional file 1.**

## Data Availability

Data can be requested from the corresponding author.

## Abbreviations

ART: Anti-retroviral therapy
PLWHIV: People living with HIV
VL: Viral load
