# Supplementary material for: Returning to care after incarceration with HIV: the French Guianese experience
Source: BMC Public Health. 2020 May 24;20:754. doi: 10.1186/s12889-020-08772-9 (PMC7245866; doi:10.1186/s12889-020-08772-9)
Supplement: Supplementary file 1 — Additional file 1. [file 12889_2020_8772_MOESM1_ESM.docx]

**Supplementary material**

**Factors associated with uninterrupted HIV care at 12+/- 3 months, after release from French Guiana correctional facility (n=132).**

|  | HIV care (%) | | Crude IRR  (95%CI) | p | Adjusted IRR (95%CI) | p |
| --- | --- | --- | --- | --- | --- | --- |
|  | Yes (n=36) | No  (n=96) |  |  |  |  |
| **Age** | | | | | | |
| Less than 30 | 11.1 | 88.9 | Reference |  | Reference |  |
| 30-40 | 21.3 | 78.7 | 1.9 (0.6-6.2) | 0.27 | 2.1 (0.7-5.7) | 0.17 |
| More than 40 | 45.4 | 54.6 | 4.1 (1.3-12.5) | 0.01 | 2.9 (1.1-7.7) | 0.03 |
|  |  |  |  |  |  |  |
| **Chronology between ART and incarceration** | | | | | | |
| - No ART on release | 7.1 | 92.9 | Reference |  | Reference |  |
| - ART started/restarted in prison | 32.1 | 67.9 | 4.5 (1.6-12.3) | <0.01 | 3.8 (1.5-10.1) | <0.01 |
| - On ART while incarcerated | 64.7 | 35.3 | 9.1 (3.7-21.9) | <0.01 | 6.9 (2.8-17.0) | <0.01 |
| **Comorbidity** | 45.5 | 54.5 | 2.5 (1.4-4.3) | <0.01 | 1.8 (1.2-2.8) | 0.01 |

*Defined by at least one visit in each interval-time (M0-M3, M3-M9, M9-M15)

Cofactors tested but not retained after final analysis (p>0·20) were : psychiatric background, addiction, duration of the index incarceration, homelessness. Cofactors retained in the final analysis (p<0.20) but not in the final model were : sex, native country, crack-use, advanced HIV-disease, chronology between 1^st^ positive HIV test and index incarceration
